# Utilization of institutional delivery and associated factors among mothers in rural community of Pawe Woreda northwest Ethiopia, 2018

**DOI:** 10.1186/s13104-019-4450-6

**Published:** 2019-07-12

**Authors:** Tewodros Eshete, Mandefro Legesse, Mulatu Ayana

**Affiliations:** 1 Department of Health Informatics, College of Health Sciences, Debre Markos University (DMU), Debre Markos, Ethiopia; 2 Public Health Emergency Management (PHEM), South Gondar Zone Health, Gondar, Ethiopia; 3 Department of Public Health, College of Health Sciences, Debre Markos University (DMU), Debre Markos, Ethiopia

**Keywords:** Institutional delivery, Pawe, Ethiopia

## Abstract

**Objective:**

In most of sub-Saharan African countries the significance of delivering in health institution and threats of death is still little known. This study is to assess utilization of institutional delivery and associated factors among mothers who gave birth in the last 12 months prior to the study in rural community of Pawe Woreda, Benishangul-Gumuz, northwest Ethiopia, 2018. A community-based cross-sectional study was conducted on 623 mothers.

**Results:**

Overall deliveries 60.5% were assisted at health facilities. Multivariable logistic regression showed that Mothers educational status, Antenatal Care visit during their recent pregnancy, delivery plan of recent pregnancy, maternal knowledge on benefit of institutional, decision power about place of delivery and distance to reach the nearby facility on were significantly associated with utilization of institutional delivery. The utilization of institutional delivery services among rural women in Pawe Woreda had improvements but still low. Intensifying women education, up taking Antenatal Care potential services, address health education for mothers about benefit of institutional birth and counseling danger sign of labor and delivery, involving couples decision power of facility birth and expanding health facilities in the community are recommended interventions.

## Introduction

Despite global progress in reducing maternal mortality, there is a need for immediate action to meet the ambitious 2030 sustainable development goals (SDG) and ultimately eliminate preventable maternal mortality [1]. In developing countries, nearly all maternal mortality (99%) occur in sub-Saharan Africa countries, in fragile, and humanitarian settings [2]. Despite a number of Sub-Saharan African countries halved their maternal mortality level since 1990, there are the most off-track achievements of continent based maternal deaths, where rural women remain the highest burden compared to those urban women accordingly [3].

A key strategy for reducing maternal mortality is the timely use of adequate, high, and quality maternal health services. Complication of delivery and mortality can be prevented through a health institution assisted care with skilled health care providers in an enabling environment and effective referral system [4–6].

According to the 2016 Ethiopian Demographic Health Survey (EDHS) the proportion of health facility births attended in Ethiopia is still very much lower than that of countries in sub-Saharan Africa (SSA) 47%. Even if women who have access to the services, the proportion of births occurring at health facilities were at national level accounts only 26% of births were took place at health institution and the progression of maternal mortality ratio accounts 412 deaths per 100,000 live births. Yet the aim of practicing facility based birth is to protect life of mothers and their babies to encourage efforts and to reduce life threatening conditions by improving care and healthy under health professional support and supervision [7–9].

In Ethiopia the proportion of institutional delivery greatly varies across the residence where the mothers are living. According to EDHS 2016, Central statistics Agency (CSA) 80% of births to urban mothers were assisted by a skilled provider and 79% were delivered in a health facility, as compared with 21% and 20%, respectively, of births to rural women. Developing regions has the lowest percentage of women whose births were delivered by a skilled provider or delivered in a health facility (16% and 15%) [9].

Although, the Federal Ministry of Health (FMOH) has applied a multi-prolonged approach to increase utilization of institutional delivery by improving access and strengthening facility-based maternal services but, the proportions of births attended by skilled personnel is only 28%, which is very much lower than that of the SSA that was around 53% [7, 9, 10]. The percentage of skilled birth attendant in Ethiopia is one of the lowest in the world. Regarding Benishangul-Gumuz region where this study is conducted, institutional delivery service utilization is low (25.7%) [9]. Therefore this study aimed to assess the utilization of institutional delivery in the study area and identify factors that can significantly determine it.

## Main text

### Methods

Community based cross sectional study was conducted on mothers who gave birth in the last 12 months prior to study in rural community of Pawe Woreda, Benishangul Gumuz, northwest Ethiopia. There were 623 mothers sampled from 17 rural “kebeles” (The smallest administrative units of Ethiopia) of Pawe Destrict (“Woreda”) using Probability Proportional to Size method.

Face to face interview was conducted by well-trained data collectors using pretested semi- structured questionnaires and this was supervised by three professional Midwife supervisors. Every day after data collection, questionnaires were reviewed and checked for completeness and relevance by the supervisors and principal investigator and the necessary feedback were offered to data collectors in on spot. Effective follow up and daily supervision were carried out.

#### Statistical analysis

The data was checked for completeness, consistencies, missing values and then coded, entered, cleaned and analyzed using Statistical Package for Social Science (SPSS) version 20. Data was presented using tables, graphs and figures. In bivariable logistic analysis, variables with P-value less than 0.25 were candidate to multivariable logistic regression analysis and P value of < 0.05 was used as cutoff point for identifying statistically significant association between independent and outcome variable at 95% confidence intervals.

### Results

A total of 623 mothers who gave birth in the last 12 months were interviewed with 97.3% response rate. Majority of the respondents (32.7%) were in the age group of 25–29 years with mean (SD) age of 28.93 ± 6.53 years. Majority 559 (89.7%) of the study subjects were married and 85.2% of mothers were housewives. In our study area, near to two-third of mothers had no formal education attended and more than half of married mothers (54.9%) reported that their husbands did not get to formal education (Table 1).

**Table 1:** Socio-demographic characteristics of respondents in Pawe Woreda, northwest Ethiopia, April 2018

Variables	Categories	Frequency	Percent
Age	15–19	24	3.9
	20–24	131	21.0
	25–29	204	32.7
	30–34	115	18.5
	> 35	149	23.9
	Mean (± SD), 28.93 ± 6.53		
Marital status	Married	559	89.7
	Divorced	46	7.4
	Widowed	12	1.9
	Single	6	1.0
Ethnicity	Amhara	536	86.0
	Oromo	10	1.6
	Kembata	46	7.4
	Others	31	5.0
Mothers occupation	House wives	531	85.2
	Merchant	22	3.5
	Private employer	40	6.4
	Others	30	4.8
Mothers educational status	Not formally educated	393	63.1
	Primary education	187	30.0
	Secondary and above	43	6.9
Husbands educational status	Not formally educated	307	54.9
	Primary education	187	33.4
	Secondary and above	65	11.7

This study found that, the minimum and maximum age at first birth of mothers were 15 and 32 respectively with mean (± SD) of 20.61 (± 3.31) years and 46.4% of respondents gave birth at the age of 20–24 years. Most (60.5%) of the respondents in our study area delivered their last child at health institution where 158 (25.4%), 128 (20.5%), and 89 (14.3%) of deliveries were attended at health posts, hospitals, and health centers respectively under the support of skilled birth attendants (Table 2).

**Table 2:** Obstetric characteristics and actual delivery of respondents in Pawe Woreda, northwest Ethiopia, April 2018

Variables	Categories	Frequency	Percent
Number of pregnancy (gravidity)	1	111	17.8
	2–4	346	55.5
	≥ 5	166	26.6
Number of births (parity)	1	118	18.9
	2–3	252	40.4
	> 3	253	40.6
Number of still births ever had	0	512	82.2
	1	81	13.0
	2–4	30	4.8
Planned pregnancy	Planned	496	79.6
	Unplanned	127	20.4
ANC visits	Yes	516	82.8
	No	107	17.2
Number of ANC visits	0	107	17.2
	1–3	310	49.8
	≥ 4	206	33.1
Place of ANC follow up	Hospital	181	29.1
	Health center	79	12.7
	Health post	255	40.9
	Others	1	0.2
Delivery plan	Yes	485	77.8
	No	138	22.2
Got information about pregnancy and delivery complication	Yes	438	70.3
	No	129	20.7
Ever given birth in health institutions	Yes	283	45.4
	No	234	37.6
Women delivery in the last birth	Health facility	377	60.5
	Home	246	39.5
Place of institutional delivery	Hospital	128	20.5
	Health center	89	14.3
	Health post	158	25.4
	Others	2	0.3
Attending PNC services	Yes	423	67.9
	No	200	32.1
Decision about place of delivery	Just me	200	32
	My husband	46	7.4
	Both my husband and me	322	51.7
	My relatives	55	8.9

The adjusted odds of giving birth at health institution was 68.7% less likely (AOR = 0.313 [95% CI 0.127, 0.768]) among mothers who had no formal education than those who had secondary education and above. The adjusted odds of giving birth at health institution was 3.6 times higher (AOR = 3.6 [95% CI 1.709, 7.59]) among mothers who attended ANC visits in recent pregnancy than those who didn’t attend the services. Mothers who had delivery plan (a pre settled plan where to delivery) of their recent pregnancy were 3.33 times more likely to deliver at health institutions (AOR = 3.33 [95% CI 1.8, 6.17]) than those counter parts. The adjusted odds of institutional delivery was 60% more likely (AOR = 1.60 [95% CI 1.043, 2.305]) among mothers who have adequate knowledge about its benefit than their counterpart. Decision power of couples about place of delivery were found to be a strong predictor. Mothers who decided together with their husband on the place of delivery were 3.5 times more likely to give birth at health institution (AOR = 3.542 [95% CI 1.790, 7.007]) than their counterpart.

Distances to health institutions appeared to be associated with deliveries at health facilities. Mothers who spent less than half of an hour to walk to health facility were thrice more likely to give birth at health institution (AOR = 2.915 [95% CI 1.823, 4.660]) than those who spent longer time (Table 3).

**Table 3:** Bivariable and multivariable analysis of factors associated with utilization of institutional delivery in Pawe Woreda, Benishangul-Gumuz, northwest Ethiopia April 2018

Variables	Utilization of institutional delivery		COR (95% CI)	AOR (95% CI)	P-value
	Yes	No			
Mother educational status					
Not formally educated	224	169	0.351 (0.164, 0.751)*	0.313 (0.127, 0.768)*	0.011
Elementary (grade 1–8)	119	68	0.463 (0.210, 1.024)*	0.479 (0.189, 1.211)	0.120
Secondary (grade 9 and above)	34	9	1	1	
ANC visit in recent pregnancy					
Yes	357	20	9.052 (5.772, 14.197)*	3.33 (1.80, 6.17)*	0.001
No	159	87			
Maternal knowledge on benefit of institutional delivery					
Adequate knowledge	210	91	2.142 (1.541, 2.977)*	1.60 (1.043, 2.305)*	0.001
Inadequate knowledge	167	155	1	1	
Decision power about place of delivery					
Just me	95	105	0.938 (0.516, 1.705)	0.942 (0.476, 1.863)	0.864
My husband	21	25	0.871 (0.397, 1.910)	0.878 (0.358, 2.149)	0.775
Both me and my husband	234	88	2.758 (1.540, 4.938)*	3.542 (1.790, 7.007)*	0.001
My relatives	27	28	1	1	
Distance to reach the nearby health facility on foot (mins)					
≤ 30	326	136	5.170 (3.509, 7.618)*	2.915 (1.823, 4.660)*	0.001
> 30	51	110	1	1	

* P < 0.25

** P < 0.05

### Discussion

This community based study has attempted to identify utilization of institutional delivery and associated factors among mothers who gave birth in the last 12 months prior to the study in rural community of Pawe Woreda, Benishangul-Gumuz, northwest Ethiopia. The study showed that utilization of institutional delivery service was 60.5% at 95% CI (57.1, 64.7) in the district. However more than one-third of mothers gave birth at home. This finding is consistence with the study conducted in Sodo district, Kenya, rural Zambia and sub-Saharan Africa where the proportion of women who gave birth on health facilities were 53%, 61%, 62.2% and 57% respectively [11–14]. But this proportion had improvements as compared with institutional delivery service utilization among rural mother at national and regional estimates [9]. Even though still below the Health Sector Transformation Plan (HSTP) of Ethiopia to increase institutional deliveries attended by skilled health personnel to 95%. The reason for this difference might be due to studies was conducted after the Ethiopian government started free delivery services at all levels of health institutions and there might have been improvements in accessibility of health center to health post cluster integration. However, the findings of this study is lower than that of studies conducted on Bench Maji zone and Debre Berhan zones which was 78.3% and 80.2% respectively [15, 16]. This difference could be due to difference in awareness and knowledge of facility birth, health education and accessibility of health facility in relation to socio- demographic characteristics.

The multivariable logistic regression analysis showed that mothers who had not formally educated were 68.7% less likely to give birth at health institution (AOR = 0.313 [95% CI 0.127, 0.768]) than those who attended secondary educated and above. This finding was also consistence with other studies done in Enderta district, Ethiopia and India [17, 18]. The possible reason might be when mothers were not more educated they might have less awareness and initiation in relation to place of delivery with communicate and understand the health status of possible follow up, fear, awareness and consultation perceived to their health status.

Mothers who visited health facilities for ANC during their recent pregnancy were more like to deliver in health institution than those who didn’t. This result was in-line with studies conducted in rural Hadya zone, Enderta district, Bahirdar, and Nigeria [17, 19–21]. This could be due to the reason that antenatal care provides mothers to get counsel about birth preparedness and complication readiness that can promote mothers to deliver at health facility with skilled attendants. Mothers who had delivery plan for their recent pregnancy were also found to be significant predictor of institutional delivery. This finding is consistence with studies done in rural Hadya zone, Guji zone, and Woldia [20, 22, 23]. The possible explanation might be awareness of preparation and seeking care during and after birth could influence maternal choice to place of delivery and enhances the good birth outcome.

Maternal knowledge on benefit of institutional delivery was found to be an independent predictor of giving birth in a health facility. Mothers who had adequate knowledge on benefit of institutional delivery were more likely to utilize health institutions as compared to those who had inadequate knowledge. This is consistent with the finding from a study done in Banja woreda and Sodo district [12, 24]. This might imply that mothers, who know the danger signs have a greater fear of complications, lead them to seek skilled attendance during birth. Moreover, if women are knowledgeable about danger signs they will have a plan to deliver in health facility to address birth complications.

Final decision power about place of delivery was found to be a strong predictor of institutional delivery. Mothers who decided with their husband where to delivery their child are more prone to deliver at health institution. This finding was also consistence with studies done in rural Hadya zone, Holeta town, and Awash Fentale [20, 25, 26]. In most situations, relatives and neighbors who are the main decision maker in the community can be one of the reasons why laboring mothers stay at home during delivery [27].

Mothers who spent less than half of an hour to walk to health facility were more likely to delivery at health. This finding is consistence with studies conducted in Ethiopia, rural India, and Awash Fentale district [10, 26, 28]. Mothers whose residence was near to the facility could have access to health education and ANC services. Moreover, mothers who resided to the nearby health facilities had no problem of transportation to attend the institutional and able to early manage obstetric problems at any time.

### Conclusions

The utilization of institutional delivery service among rural women in Pawe Woreda had improvements as compared to the national and regional estimates, but it is still below the HSTP of Ethiopia which plan to increase deliveries attended by skilled health provider to 95%. This study identified that mothers’ educational status, ANC visit in recent pregnancy, delivery plan of recent pregnancy, maternal knowledge on benefit of institutional delivery, decision power about place of delivery, and distance to reach to the nearby facility on foot were significantly associated with utilization of institutional delivery.

Intensifying women education, encouraging focused Antenatal care potential services, empowering women to have delivery plan for birth preparedness and complication readiness, provision of health education to fill the gap of maternal knowledge on benefit of institutional delivery, promoting couples involvement in decision making and expanding health facilities are recommended interventions to increase institutional delivery and prevent the obstetric consequence of home delivery. Policy makers has to focus on promoting and increasing institutional delivery. Every responsible stakeholders have to be ready to take their share on the recommendations.

#### Strength

Our study tried to look as many variables as possible.

## Limitation

Some variables (e.g. age at first birth) may face recall bias.

## Data Availability

Supporting data for the current study are available from the corresponding author on reasonable request.

## Abbreviations

ANC: ante natal care
AOR: adjusted odds ratio
CI: confidence interval
COR: crudes odds ratio
OR: odds ratio
SSA: sub-Saharan Africa
